# Optimization-driven distribution of relief materials in emergency disasters

**DOI:** 10.1007/s40747-021-00290-4

**Published:** 2021-02-10

**Authors:** Yan Yan, Xinyue Di, Yuanyuan Zhang

**Affiliations:** 1 Shenyang University of Technology, Shenyang, China; 2 School of Public Health, Dalian Medical University, Dalian, China

**Keywords:** Relief materials, Disasters, Distribution, Optimization

## Abstract

The distribution of relief materials is an important part of post-disaster emergency rescue. To meet the needs of the relief materials in the affected areas after a sudden disaster and ensure its smooth progress, an optimized dispatch model for multiple periods and multiple modes of transportation supported by the Internet of Things is established according to the characteristics of relief materials. Through the urgent production of relief materials, market procurement, and the use of inventory collection, the needs of the disaster area are met and the goal of minimizing system response time and total cost is achieved. The model is solved using CPLX software, and numerical simulation and results are analyzed using the example of the COVID-19 in Wuhan City, and the dispatching strategies are given under different disruption scenarios. The results show that the scheduling optimization method can meet the material demand of the disaster area with shorter time and lower cost compared with other methods, and can better cope with the supply interruptions that occur in post-disaster rescue.

## Introduction

With the continuous diversification and rapid development of the global economy, sudden events worldwide have frequently occurred and gradually escalated, seriously threatening the safety of people's lives and properties [2, 4, 5]. Sudden events are emergencies which include natural disasters, accidents, public health threats, and social security events that occur suddenly leading to serious harm and, thus, require emergency response measures to be taken in response [8–10]. Examples of such emergencies include the global COVID-19 in 2020, the fire in Australia in 2019, the leakage of the Fukushima nuclear power plant in Japan in 2011, and the September 11 incident in the United States in 2001. Each disaster has brought great economic losses and social impacts on the world. For instance, data from the Risk Research Center of the Judge Business School of Cambridge University show that the COVID-19 could cause a global economic loss of US$82 trillion within 5 years and the worldwide death toll from the epidemic has now reached 650,000.

In the case of frequent sudden events, the government is required to dispatch relief materials to disaster areas for rescue. Relief materials refer to the material guarantee necessary for the entire response to emergencies [11, 13, 14]. The dispatch of emergency supplies often faces many difficulties; for example, paralysis of logistic nodes and paths by the disasters, shortages in the necessary emergency supplies, and the inefficient government's resource scheduling activities [15, 17, 19]. Therefore, how to dispatch relief materials reasonably, meet the needs of the disaster area timely, minimize operation costs, and ensure the smooth progress of post-disaster rescue work are problems that scholars need to study in-depth.

## Literature review

The research on emergency logistics in sudden events is currently relatively mature, mainly divided into basic theory of emergency logistics, materials’ distribution and transportation optimization in emergency logistics, and construction of emergency logistics systems [20, 22–24]. The scope of this research paper is limited to material distribution and transportation optimization.

Optimal transportation scheduling and material distribution are important links in emergency logistics and the key to realizing its timeliness. In other studies, various experts such as Knott [12] and Eldessouki [6] have studied the transportation scheduling of relief materials under different constraints with the aim of achieving the minimum transportation cost. Linet et al. [16] established an emergency rescue material transportation model based on mixed-integer programming and conducted an in-depth study on a multi-stage and multi-objective disaster relief material distribution problem; however, the model discussed the transportation process more comprehensively instead, and thus, it still has obvious limitations. Zhu Hongli et al. [25] proposed a dispatch-rescheduling model under the pre-disaster selection and post-disaster demand interference of the distribution center according to the characteristics of the dynamic changes in material demand. Fu Jiangyue et al. [7] established the model of post-disaster distribution materials. The goal of this model is to maximize the demand satisfaction rate of the disaster site, and the efficiency goal is to minimize the delivery time of emergency supplies. In the actual earthquake rescue, the impact of the disaster victims' psychology on the formulation of relief material distribution plans cannot be ignored; however, the current research on the disaster victims' psychology is still in its infancy. Wang Xuping et al. [21] considered the irrational comparison psychology of the victims to the quantity and arrival time of relief materials, and established a relief material distribution model; Song Yinghua et al. [18] established an optimal scheduling model for relief materials, which solved the problems of the location of the distribution centers and multimodal transportation. The goal of this model is to maximize the satisfaction of victims' perception time and material quantity and minimize the total cost. In addition, the research on the distribution of materials under partial damage is relatively simple. Alem et al. [1] constructed a two-stage stochastic planning model for uncertainties such as random supply and donation and road network damage. The first stage is site selection and reserve decision-making, and the second stage is the allocation of available materials. Baharmand et al. [3] considered the impact of delivery delays, material loss, decay, and other transportation risks on relief material scheduling, and studied distribution scheduling planning.

The above studies mostly consider path optimization and material distribution issues and rarely involve some supply interruptions in sudden events. Therefore, this article addresses the above issues and considers supply interruptions in the distribution of emergency logistics, and divides the interruptions into node disruption, path disruption, and node and path disruption simultaneously. On this basis, an optimal scheduling model is constructed and proposed.

## Methodology

### Problem description

When a sudden catastrophic event occurs, the disaster area could fall into a shortage of rescue materials at any time. At this time, other regions are required to temporarily support the supply of relief materials to the affected area. The overall arrangement of the material dispatch constitutes the content of this article.

**Fig. 1:**
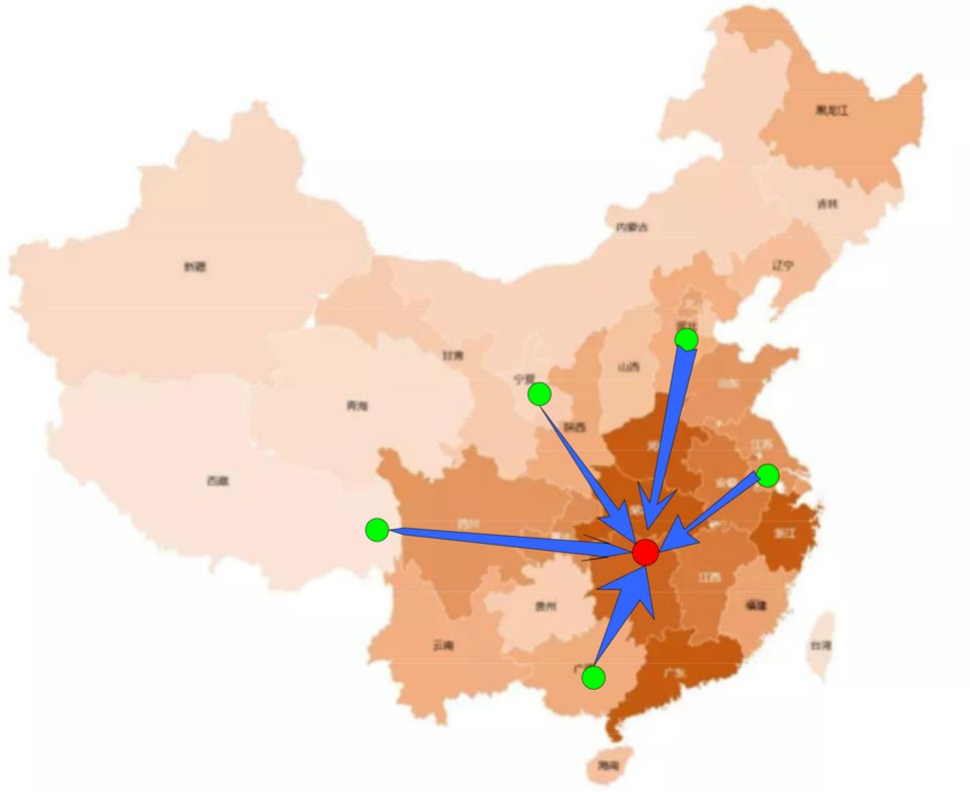
Transportation of emergency medical materials during the epidemic

In December 2019, the COVID-19 that swept the world broke out in Wuhan, China. At the beginning of the outbreak, the situation in Wuhan was more severe than that of other cities that are far away from Wuhan. During that time, Wuhan was facing a high shortage of N95 masks and medical protective clothing. Therefore, the country urgently needed to import masks and protective clothing from areas with fewer epidemics giving priority to Wuhan. At the same time, medical material manufacturers across the country have expedited their production and continuously transported the materials to various hospitals (as shown in Fig. 1).

Since it was the Spring Festival, the majority of the manufacturers were on holiday. The epidemic prevented people from gathering, and various air routes were suspended. Under such circumstances, it is difficult to expedite the supply and smooth transportation of medical materials to Wuhan city. The consequences are as follows:

1. The node disruption (inability of manufacturers to resume production): After the Spring Festival, the COVID-19 caused the holiday to be extended. Workers were afraid of contracting COVID-19 and dare not return to work from their hometown. Workers who can resume work cannot travel because of traffic control. Therefore, many medical material manufacturers cannot produce in time.
2. The path disruption (inability of existing medical supplies to be delivered to the disaster area). Medical supplies are collected through market purchases and inventories from the cities where the epidemic is milder. However, it is difficult to transport these materials to Wuhan, because many air and land transportation routes have been interrupted.

#### Hypothesis

**Fig. 2:**
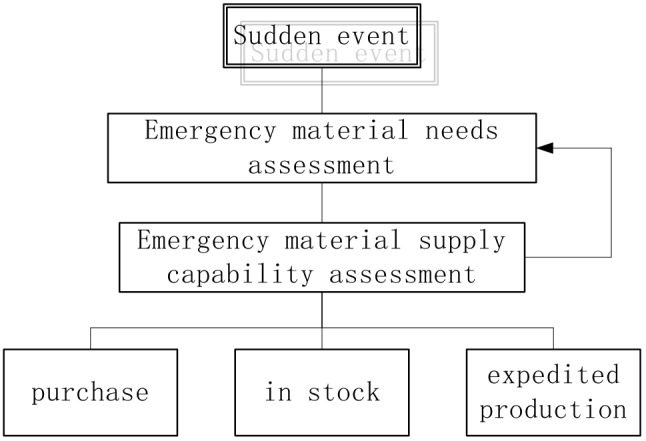
Relief materials’ guarantee system

1. Though there are many types of medical materials, the medical materials in this article refer to N95 masks and medical protective clothing. These two materials have a long shelf life and there is no problem with product quality declination over time.
2. *I* cities are supporting Wuhan, and there are three ways to obtain medical supplies in each city, namely, market procurement, use of inventory, and expedited production. These are as shown in Fig. 2. When the ith node transports materials to the disaster area, priority is given to the use of inventory and market-purchased medical materials. When these materials fail to meet the needs of the disaster area, urgent production is carried out locally.
3. There are many routes to transport materials: air, land (automobile), sea, etc. Since Wuhan is an inland city, to simplify the calculation, this model only considers air and land transportation.
4. In view of the development and application of the Internet of Things technology, in the emergency dispatch network, the total demand for materials in the disaster area and the supply capacity of each node to provide assistance are known.

### Symbol description

Parameters:

O: Disaster point.

$t$: Planning time period $t = 1,2, \ldots ,T$.

$i$: There are $i$ support points transporting materials to point O, $i \in I$.

$w$: Different modes of transportation, $w \in W$.

$L_{i}$: Distance from the $i$th support point to the disaster area O.

$v_{wi}$: Transportation rate of the route $i - O$ using $w$ transportation mode.

${\text{gc}}$: Unit cost of goods.

${\text{ec}}$: Expedited production cost per unit product.

${\text{rc}}_{w}$: Transportation cost of unit product using $w$ transportation mode.

$D$: Total material demand at point O.

$B_{i}$: Upper limit of the total supply of $i$-th node.

$h$: Time required to expedite the production of unit products.

$U_{{{\text{ot}}}}$: Inventory of medical materials at point O at the end of t period.

$S_{{{\text{ot}}}}$: Demand for medical supplies at point O at the end of t period.

Decision variables:

$X_{{{\text{wit}}}}$: Amount of expedited production materials transported by means of $w$ on the $i - O$ path during $t$ period.

$Y_{{{\text{wit}}}}$: Amount of directly purchased materials transported by means of $w$ on the $i - O$ path during $t$ period.

$Z_{{{\text{wit}}}}$: Amount of materials used in inventory transported by means of $w$ on the $i - O$ path during $t$ period.

$\partial_{{{\text{wit}}}}$: 0–1 variable, if there are expedited production materials on the $i$th node during $t$ period, $\partial_{{{\text{wit}}}} = 1$, otherwise, $\partial_{{{\text{wit}}}} = 0$.

$\beta_{{{\text{wit}}}}$: 0–1 variable, if there are directly purchased materials on the $i$th node during $t$ period, $\beta_{{{\text{wit}}}} = 1$, otherwise, $\beta_{{{\text{wit}}}} = 0$.

$\varepsilon_{{{\text{wit}}}}$: 0–1 variable, if there are materials used in inventory on the $i$th node during $t$ period, $\varepsilon_{{{\text{wit}}}} = 1$, otherwise, $\varepsilon_{{{\text{wit}}}} = 0$.

$\theta_{{{\text{wit}}}}$: 0–1 variable, when materials are transported by means of transportation $w$ on the $i - O$ path during $t$, $\theta_{{{\text{wit}}}} = 1$, otherwise, $\theta_{{{\text{wit}}}} = 0$.

### Mathematical model

Multi-objective programming is a branch of mathematical programming that studies the optimization of more than one objective function in a given area. This study chooses the multi-objective planning method to solve the problem. Emergency dispatch has a weak economy, and the main purpose of introducing cost factors in this article is to evaluate the optimization effect of the system in many aspects. Therefore, this paper takes the shortest total time and the minimum total cost of emergency dispatch as the optimization goals, and establishes the following multi-objective planning model:

Objective function:

1 $$ \min :\left\{ \begin{gathered} \sum\limits_{t = 1}^{T} {\sum\limits_{i = 1}^{I} {\sum\limits_{w = 1}^{W} {\partial_{{{\text{wit}}}} X_{{{\text{wit}}}} } } } \left( {gc_{t} + ec_{t} + \theta_{{{\text{wit}}}} rc_{{{\text{wt}}}} } \right) + \sum\limits_{t = 1}^{T} {\sum\limits_{i = 1}^{I} {\sum\limits_{w = 1}^{W} {\beta_{{{\text{wit}}}} Y_{{{\text{wi}}}} } } } \left( {gc_{t} + \theta_{{{\text{wit}}}} rc_{{{\text{wt}}}} } \right) \hfill \\ + \sum\limits_{t = 1}^{T} {\sum\limits_{i = 1}^{I} {\sum\limits_{w = 1}^{W} {\varepsilon_{{{\text{wit}}}} Z_{{{\text{wit}}}} } } } \left( {gc_{t} + \theta_{{{\text{wit}}}} rc_{{{\text{wt}}}} } \right) \hfill \\ \end{gathered} \right\} $$

2 $$ \min :\left\{ {\sum\limits_{t = 1}^{T} {\sum\limits_{i = 1}^{I} {\sum\limits_{w = 1}^{W} {\partial_{{{\text{wit}}}} X_{{{\text{wit}}}} } } } h + \sum\limits_{t = 1}^{T} {\theta_{{{\text{wit}}}} } \frac{{L_{i} }}{{V_{{{\text{wit}}}} }}} \right\}. $$

Restrictions:

3 $$ \sum\limits_{t = 1}^{T} {\sum\limits_{i = 1}^{I} {\sum\limits_{w = 1}^{W} {\left( {\partial_{{{\text{wit}}}} X_{{{\text{wit}}}} + \beta_{{{\text{wit}}}} Y_{{{\text{wit}}}} + \varepsilon_{{{\text{wit}}}} Z_{{{\text{wit}}}} } \right)} } } \ge D $$

4 $$ \sum\limits_{t = 1}^{T} {\sum\limits_{i = 1}^{I} {\sum\limits_{w = 1}^{W} {\partial_{{{\text{wi}}}} X_{{{\text{wi}}}} } } } t + \sum\limits_{t = 1}^{T} {\theta_{{{\text{wit}}}} \frac{{L_{i} }}{{V_{{{\text{wit}}}} }}} \le T $$

5 $$ \sum\limits_{t = 1}^{T} {\sum\limits_{w = 1}^{W} {\left( {X_{{{\text{wit}}}} \partial_{{{\text{wit}}}} + Y_{{{\text{wit}}}} \beta_{{{\text{wit}}}} + Z_{{{\text{wit}}}} \varepsilon_{{{\text{wit}}}} } \right)} } \le B_{i} $$

6 $$ U_{o(t - 1)} + \sum\limits_{i = 1}^{I} {\sum\limits_{w = 1}^{W} {\left( {\partial_{{{\text{wit}}}} X_{{{\text{wit}}}} + \beta_{{{\text{wit}}}} Y_{{{\text{wit}}}} + \varepsilon_{{{\text{wit}}}} Z_{{{\text{wit}}}} } \right)} } = S_{ot} + U_{ot} $$

7 $$ \partial_{wi} \left\{ {\begin{array}{*{20}c} {{1}\quad {\text{If there are expedited production transported by }}w {\text{mode on the}}\;i - O\;{\text{path}}} \\ {0\quad {\text{or else}}} \\ \end{array} } \right. $$

8 $$ \beta_{wi} \left\{ {\begin{array}{*{20}c} {1\quad {\text{If there are directly purchased materials transported by }}w{\text{ mode on the}}\;i - O\;{\text{path}}} \\ {0\quad {\text{or else}}} \\ \end{array} } \right. $$

9 $$ \varepsilon_{wi} \left\{ {\begin{array}{*{20}c} {1\quad {\text{If there are materials used in inventory transported by }}w{\text{ mode on the}}\;i - O\;{\text{path}}} \\ {0 \quad {\text{or else}}} \\ \end{array} } \right. $$

10 $$ X_{{{\text{wit}}}} ,Y_{{{\text{wit}}}} ,Z_{{{\text{wit}}}} \ge 0\quad \forall w \in W,\;i \in I,\;t \in T. $$

Formulas (1) and (2) are objective functions. Formula (1) is the total cost of materials. The total cost consists of three parts: the cost of urgent production of materials, the cost of materials purchased directly, and the cost of materials from the inventory. $\sum\nolimits_{t = 1}^{T} {\sum\nolimits_{i = 1}^{I} {\sum\nolimits_{w = 1}^{W} {\partial_{{{\text{wit}}}} X_{{{\text{wit}}}} } } } \left( {{\text{gc}}_{t} + {\text{ec}}_{t} + \theta_{{{\text{wit}}}} {\text{rc}}_{{{\text{wt}}}} } \right)$ is the total cost of expedited production materials on all routes. $\sum\nolimits_{t = 1}^{T} {\sum\nolimits_{i = 1}^{I} {\sum\nolimits_{w = 1}^{W} {\beta_{{{\text{wit}}}} Y_{{{\text{wi}}}} } } } \left( {{\text{gc}}_{t} + \theta_{{{\text{wit}}}} {\text{rc}}_{{{\text{wt}}}} } \right)$ is the total cost of materials purchased directly. $\sum\nolimits_{t = 1}^{T} {\sum\nolimits_{i = 1}^{I} {\sum\nolimits_{w = 1}^{W} {\varepsilon_{{{\text{wit}}}} Z_{{{\text{wit}}}} } } } \left( {{\text{gc}}_{t} + \theta_{{{\text{wit}}}} {\text{rc}}_{{{\text{wt}}}} } \right)$ is the total cost of materials used in inventory. Formula (2) is the total time for materials to reach the disaster area, including expedited production time and transportation time.

Among the constraints, formula (3) indicates that the total supply of materials must meet all the needs of the disaster area. Formula (4) represents the delivery time limit for the materials to meet the needs of the disaster area. Formula (5) is the supply capacity constraint of each point. Formula (6) is the balance constraint of logistics in each period. Formulas (7–9) are 0, 1 variable, and formula (10) is a non-negative constraint.

### Model solving

There are many ways to solve the multi-objective programming problem. This article adopts the common linear weighted sum function method, and introduces the time weight coefficient $\alpha_{1}$ and cost weight coefficient $\alpha_{2}$, where $\alpha_{1} + \alpha_{2} = 1$. The values of $\alpha_{1}$ and $\alpha_{2}$ are flexibly adjusted according to the different requirements of the time and cost targets at different stages of the rescue. In the initial stage of the rescue, the shortest emergency response time is generally the main goal, $\alpha_{1}$ > $\alpha_{2}$. At the same time, to avoid the influence of the different dimensions of the time target and the cost target, the time cost coefficient $\lambda_{t}$ is introduced. This problem is now transformed into a single objective mixed-integer linear programming problem (MILP). The objective function is as follows:

11 $$ \min :\left\{ \begin{gathered} \alpha_{1} \left[ {\sum\limits_{t = 1}^{T} {\sum\limits_{i = 1}^{I} {\sum\limits_{w = 1}^{W} {\partial_{{{\text{wit}}}} X_{{{\text{wit}}}} } } } h\lambda_{t} + \sum\limits_{t = 1}^{T} {\lambda_{t} \theta_{{{\text{wit}}}} } \frac{{L_{i} }}{{V_{{{\text{wit}}}} }}} \right] + \hfill \\ \alpha_{2} \left[ {\sum\limits_{t = 1}^{T} {\sum\limits_{i = 1}^{I} {\sum\limits_{w = 1}^{W} {\partial_{{{\text{wit}}}} X_{{{\text{wit}}}} } } } \left( {{\text{gc}}_{t} + {\text{ec}}_{t} + \theta_{{{\text{wit}}}} {\text{rc}}_{{{\text{wt}}}} } \right) + \sum\limits_{t = 1}^{T} {\sum\limits_{i = 1}^{I} {\sum\limits_{w = 1}^{W} {\beta_{{{\text{wit}}}} Y_{{{\text{wi}}}} } } } \left( {{\text{gc}}_{t} + \theta_{{{\text{wit}}}} {\text{rc}}_{{{\text{wt}}}} } \right) + \sum\limits_{t = 1}^{T} {\sum\limits_{i = 1}^{I} {\sum\limits_{w = 1}^{W} {\varepsilon_{{{\text{wit}}}} Z_{{{\text{wit}}}} } } } \left( {{\text{gc}}_{t} + \theta_{{{\text{wit}}}} {\text{rc}}_{{{\text{wt}}}} } \right)} \right] \hfill \\ \end{gathered} \right\}. $$

This problem is an integer programming problem. The operating environment is Inter(R)Core(TM) i5-9500T CPU 2.20 GHZ, memory 8.00G, and the OPL language is used to generate ILOG CPLEX12.9 to implement the model. The average time is less than one (1) minute. The data in this article are obtained from the Public Health Emergency and Major Epidemic Prevention and Control Command and Dispatching Center in Wuhan, China.

## Result

**Fig. 3:**
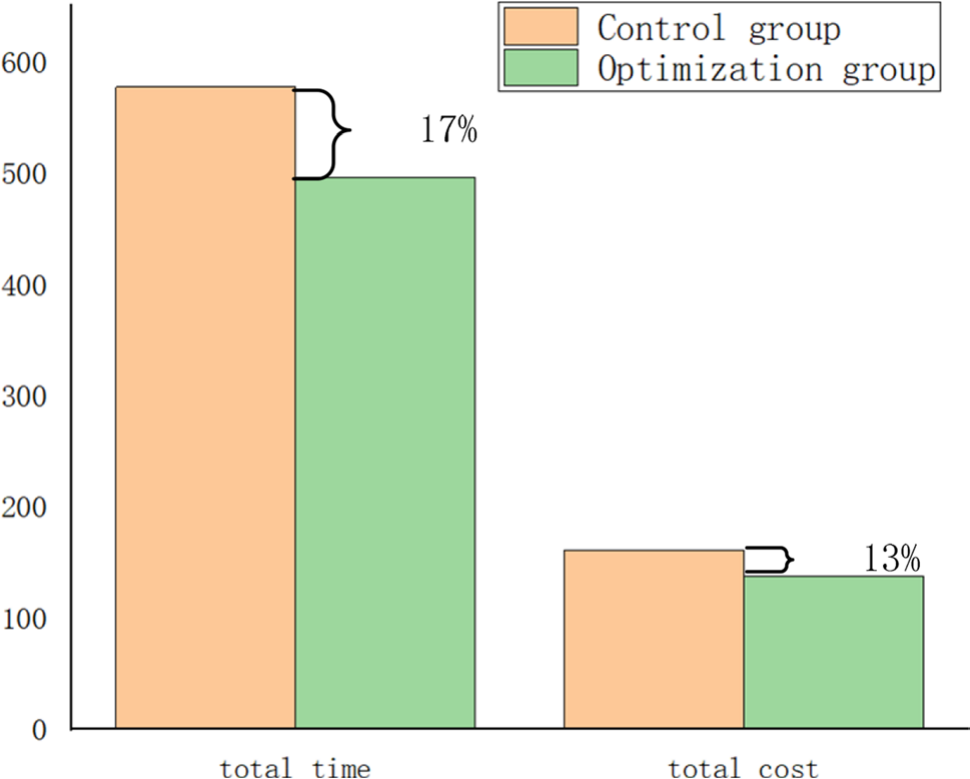
Comparison of time and cost of relief materials

**Fig. 4:**
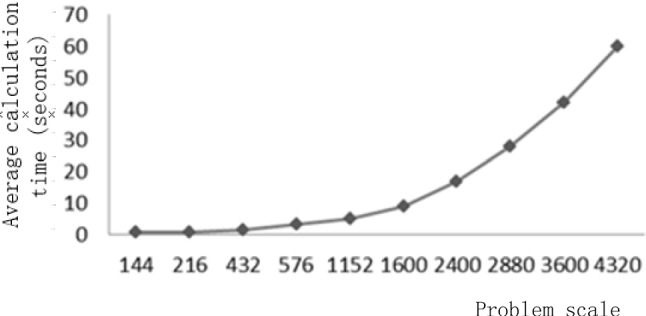
The problem scale and computation time

In this paper, a network consisting of a disaster area (O) and five (5) support points $\left( {I_{1} ,I_{2} ,I_{3} ,I_{4} ,I_{5} } \right)$ is used as the background to solve the optimization model. Comparing the optimization results with the actual scheduling results during the epidemic, the total scheduling time was reduced by 17% and the scheduling cost was reduced by 13%. The effectiveness of the model is thus verified. This is as shown in Fig. 3. The comparison of the optimization results under different time weighting factors is presented in this article. In addition, a large number of numerical experiments were carried out by taking random numbers of demand, and the percentage of time optimization rate was maintained between 15 and 20%, while the total cost optimization rate was between 10 and 15%, as shown in Table 1. Figure 4 shows the relationship between the optimization model and the computation time. As the complexity of the problem increases, the computation time gradually increases.

**Table 1:** Numerical results of optimal rate under different time weights

$\alpha$	Total time (%)	Total cost (%)
0.6	17	12
0.7	19	15
0.8	15	10
0.9	16	14

To deal with sudden events, this model considers three supply interruptions, namely, node disruption, path disruption, node, and path disruption simultaneously. The case of node disruption is taken as an example. Assuming that points $I_{1}$ and $I_{4}$ fail in the expedited production function during periods $t_{1} \sim t_{2}$ and $t_{1} \sim t_{3}$, respectively, then in the period $t_{1}$, materials’ production is carried out at points $I_{2}$, $I_{3}$, $I_{5}$, and the materials from $I_{5}$ are transported by land, $I_{1}$ and $I_{3}$ are transported by air, and $I_{2}$ is transported by a combination of air and land. During the $t_{2}$ period, only $I_{3}$ delivers materials to Wuhan, and the materials purchased and stocked are delivered by land transportation. During the $t_{{3}}$ period, only $I_{2}$ delivers materials to Wuhan using three supply methods (purchasing + inventory + production) to reach the disaster area by land transportation. During the $t_{4}$ period, $I_{4}$ is supplied in only one way, by air transportation. During the $t_{5}$ period, nodes $I_{2}$, $I_{3}$, and $I_{4}$ deliver materials to Wuhan, and no material production is required at $I_{4}$. The materials at these nodes are all transported by land. During the $t_{6}$ period, nodes $I_{1}$ and $I_{2}$ deliver materials to Wuhan, and point $I_{1}$ does not require expedited production, and all materials are transported by land (Tables 2 and 3).

**Table 2:** Scheduling scheme when nodes and paths are interrupted separately

		Node disruption				Path disruption				
Disruption scenario		$$I_{1}$$$$t_{1} \sim t_{2}$$ Expedited production disruption $$I_{4}$$ $$t_{1} \sim t_{3}$$ Expedited production disruption				Path $$I_{2} \sim O$$ Air transport disruption Path $$I_{5} \sim O$$ Land transportation disruption				
	Time period	Procurement + inventory		Expedited production		Time period	Procurement + inventory		Expedited production	
		Path	Transport	Path	Transport		Path	Transport	Path	Transport
Scheduling strategy	1	I_1_-O	Air			1	I_5_-O	Air	I_5_-O	Air
		I_2_-O	Land	I_2_-O	Air		I_1_-O	Air	I_1_-O	Air
		I_3_-O	Air	I_3_-O	Air		I_4_-O	Air	I_4_-O	Air
		I_5_-O	Land	I_5_-O	Land		I_2_-O	Land	I_2_-O	Land
	2	I_3_-O	Land			2	I_1_-O	Land	I_1_-O	Land
	3	I_2_-O	Land	I_2_-O	Land		I_2_-O	Land	I_2_-O	Land
	4	I_4_-O	Air	I_4_-O	Air		I_3_-O	Land		
	5	I_3_-O	Land			3	I_2_-O	Land	I_2_-O	Land
		I_4_-O	Land	I_4_-O	Land	4	I_1_-O	Land		
		I_2_-O	Land	I_2_-O	Land	5	I_3_-O	Air		
	6	I_2_-O	Land	I_2_-O	Land		I_5_-O	Air	I_5_-O	Air
		I_1_-O	Land			6	I_4_-O	Land	I_4_-O	Land
Demand satisfaction	100%					100%

**Table 3:** Scheduling scheme for simultaneous disruption of nodes and paths

		Node and path disruption at the same time			
Disruption scenario		Node $I_{3}$ $t_{1} \sim t_{2}$ Expedited production disruption path $I_{1} \sim O$ Air transport disruption			
	Time period	Procurement + inventory		Expedited production	
		Path	Transport	Path	Transport
Scheduling strategy	1	I_1_–O	Land	I_1_-O	Land
		I_2_–O	Air	I_2_–O	Air
		I_3_–O	Land		
	2	I_4_–0	Land	I_4_–O	Land
		I_2_–O	Land	I_2_–O	Land
	3	I_5_–O	Air	I_5_–O	Air
		I_4_–O	Land	I_4_–O	Land
		I_1_–O	Land	I_1_–O	Land
	4	I_3_–O	Land	I_3_–O	Land
		I_2_–O	Air	I_2_–O	Air
		I_5_–O	Air	I_5_–O	Air
	5	I_1_–O	Land	I_1_–O	Land
	6	I_3_–O	Air	I_3_–O	Air
Demand satisfaction	100%				

## Conclusion

A relief material distribution model for a disaster point and multiple support points is proposed. There are three sources of emergency supplies: urgent production, inventory, and market procurement. These three sources may face insufficient supply at any time. The distribution process requires both nodes and paths to be achieved. However, nodes (support points) and routes (air and land) are also at risk of disruption at any time. The model considers the situations where the node and the path are disrupted separately and simultaneously and the relief material distribution plan under different disruption combinations is given. The program can use existing conditions to meet the emergency supply needs of the disaster-affected area. Through comparison, it is found that the results obtained by the proposed model are more optimized than the actual results (when an emergency occurs) in terms of cost and time. This model can be applied to other similar emergency rescue approach.

Nevertheless, this article also has certain shortcomings. For example, the emergency supplies in this article refer to supplies such as masks and protective clothing, which have a long shelf life and do not involve quality declination during the scheduling process. However, to transport certain materials such as blood plasma, emergency food, and others, their quality and perishable characteristics must be considered during the transportation process, and the requirements for the transportation time and transportation process will be higher. In the follow-up research, we will consider the perishable characteristics of emergency supplies in the model. Setting up a backup emergency relief material center near disaster-prone areas is also one of the future research directions.
